# The Future of Bt Proteins: From Pore Formation and Insect Resistance to the Next Generation of Pest Control

**DOI:** 10.3390/toxins17110522

**Published:** 2025-10-23

**Authors:** Mario Soberón, Alejandra Bravo

**Affiliations:** Instituto de Biotecnología, Universidad Nacional Autónoma de México [UNAM], Cuernavaca 62210, Morelos, Mexico; mario.soberon@ibt.unam.mx

The remarkable success of *Bacillus thuringiensis* [Bt] in pest control worldwide resides not only on the extraordinary potency of its pesticidal proteins, but also on their narrow insect specificity, their safety for humans, and biodegradability. Together, these features make Bt-based strategies both highly effective, and ecologically sustainable. Yet, the future toward novel applications and the management of emerging resistance still demands sustained and intensive research in the coming years.

Bt produces an impressive diversity of pesticidal proteins. Hundreds of proteins have been described so far and grouped into distinct families [Cry, Cyt, Vip, Sip, Mpp, Tpp, App, and Xpp], each with a unique structure, mode of action, and insect specificity [1]. Among these, the three-domain Cry toxins are the most widely studied, typically forming parasporal crystals during bacterial sporulation process. Cyt proteins [cytolysins] play a pivotal role in mosquito control through their synergistic interaction with some Cry toxins. These interactions increase potency and are thus fundamental for future development of more potent and directed strategies to control insect pests and delay resistance of Cry toxins. Recently, it was shown that Cyt1A-like facilitates the activity three new Bt toxins (Cry53-like, Cry56A-like, and Tpp36-like), confirming that Cyt1A-like was also able to activate proteins that have a different structure compared to the classic three-domain Cry proteins [2]. Vip proteins [vegetative insecticidal proteins] include two groups of completely different proteins: the first group are the former Vip1/Vip2 proteins, now named Vpa1/Vpa2, which act as binary toxins mainly against coleopteran larvae, and the second group are the Vip3 proteins, which are highly effective against Lepidoptera and have becoming more important now, due to their successful applications in insect pest control, against agricultural pests, including their expression in transgenic crops, where combination of Cry and Vip proteins have been extremely successful. Synergistic interactions among Cry and Vip proteins have also been documented [3]. Moreover, Cry1Ab+Vip3Aa expression in maize event presented improved toxicity against *Mythimna separata* and *Paralipsa gularis* compared to individual toxins [4,5]. Some of the Sip [Secreted Insecticidal Protein] target coleopterans, and Mpp proteins [formerly ETX/Mtx2-like] show nematocidal and coleopteran activity, as recently demonstrated for Mpp23Aa/Xpp37Aa, which showed the highest toxicity against the main cotton pest, *Anthonomus grandis*, compared to all other Bt pesticidal proteins that have been tested before [6]. Tpp proteins [formerly Bin-like] are crucial in Diptera control. App proteins can synergize with Cry toxins, and the recently identified Xpp family consists of small proteins discovered through genome mining [1]. High-throughput screening programs still continue worldwide, searching for novel proteins with activity against economically important insect pests, especially those that have developed resistance to the currently used Bt toxins.

Beyond Bt itself, new pesticidal proteins are being identified from other organisms, including fungi, other bacteria, and even plants. A striking example is the family of fern-derived insecticidal proteins which structurally resemble Cry proteins [7]. Despite their low sequence identity, these proteins share high structural similarity in domains I and II of Cry toxins and are active against insects resistant to Cry1Fa or Cry2A toxins [7]. Since domain I in Cry proteins mediates pore formation and domain II participates in receptor recognition, it is proposed that these fern proteins act through a Cry-like mechanism, perforating the membranes of insect midgut cells while engaging distinct receptors. Based on these findings, it is anticipated that many more novel pesticidal proteins will be described in the future.

Resistance mechanisms to Bt pesticidal proteins remain a central challenge. To date, the most common mechanism of Cry resistance in insects involves alterations in gut receptor proteins, which disrupt toxin binding [8]. Identifying the receptors for Bt proteins and clarifying how binding is lost during resistance evolution remains a critical concern for many pesticidal proteins. Long-term monitoring efforts, such as the seven-year survey of Asian corn borer susceptibility to Cry1Ab and Cry1F, have highlighted the importance of proactive resistance surveillance to guide management strategies [9].

Additionally, in the near future, the conformational changes triggered after proteolytic activation of Bt toxins or after receptor interaction must be resolved in molecular detail to guide rational design of next-generation toxins. For example, in Vip3Aa, domain III was shown to be the primary binding domain, and proteolytic activation of this protoxin was demonstrated to trigger a structural rearrangement that is essential for receptor interaction [10]. However, util now, the specific midgut proteins participating as receptors for Vip3Aa remain unknown, and multiple groups are actively pursuing this target.

Another underexplored area concerns pore-forming domains of Bt proteins and how they interact with membranes. High-resolution structural data [cryo-EM, X-ray crystallography] of Cry and Vip toxins interacting with target membranes are urgently needed, along with structural data of toxin–receptor complexes and conformational changes after activation. Such knowledge would allow us to predict how receptor mutations confer resistance and to engineer toxins with novel binding capabilities, as well as toxins with improved pore formation activity and higher toxicity.

The diversity of Cry receptors adds further complexity to the description of Cry toxin mechanisms of action. Multiple proteins, including aminopeptidases, alkaline phosphatases, cadherins [CAD], and ATP-binding cassette transporters [ABC transporters], have been identified as Cry receptors [11]. Mutations in CAD or ABC transporters have been directly linked to field-evolved resistance, while cross-resistance patterns highlight the interconnection of receptor-mediated susceptibility [12]. CRISPR-based knockouts in lepidopteran insects have proven powerful in validating receptor contributions and identifying resistance determinants. Intriguingly, recent CRISPR studies in mosquitoes showed that loss-of-function mutations obtained by CRISPR/Cas9 in several ABC transporters did not confer resistance to several Cry proteins active against mosquitoes [13], suggesting that a more complex receptor network underlies Cry toxicity in dipteran vectors.

Interestingly, CRISPR knockout studies of different Cry receptors such as ABCC2, ABCC3, and CAD in several lepidopteran species have clearly revealed functional redundancy. ABCC2 and ABCC3 often perform overlapping roles, whereas CAD act synergistically with ABC transporters in other cases [14]. Moreover, in species such as *Ostrinia furnacalis* and *Mythimna separata*, receptor knockouts have demonstrated that Cry1Ab and Cry1Ac toxins can act through two independent toxic pathways: one requiring only ABCC2, and another depending on both ABCC3 and CAD [15,16]. In contrast, for Cry1Fa in *O. furnacalis*, only a single pathway involving ABCC2 has been observed [15]. These findings carry profound implications for resistance management in transgenic plants, as Cry toxins operating through dual pathways, like Cry1Ab and Cry1Ac, have the potential to significantly delay resistance evolution in the field [9,17].

Finally, we must not neglect the role of insect immune defenses in shaping Bt effectiveness and resistance evolution. The cellular events that occur after pore formation are still poorly understood. Emerging evidence indicates that midgut cells can internalize Cry toxin following pore formation activity and mount complex responses, such as secreting large extracellular vesicles into the gut lumen as a detoxification strategy [18]. These vesicles contain Bt pesticidal proteins as well as damaged cellular material, yet their significance in resistance evolution remains largely unexplored.

In sum, Bt pesticidal proteins represent one of the most successful biotechnological innovations in agriculture and in controlling the vectors of human diseases. However, their future impact depends on increasing our understanding of receptor interactions, resistance pathways, structural dynamics, and insect defense responses. Only by integrating this knowledge, including molecular, structural, and ecological perspectives, can we design the next generation of Bt-based solutions to combat resistance and secure the role of Bt toxins in sustainable pest management.

## References

[1] Crickmore N., Berry C., Panneerselvam S., Mishra R., Connor T.R., Bonning B.C. (2021). A structure-based nomenclature for *Bacillus thuringiensis* and other bacteria-derived pesticidal proteins. J. Invertebr. Pathol..

[2] Lai L., Villanueva M., Muruzabal-Galarza A., Fernández A.B., Unzue A., Toledo-Arana A., Caballero P., Caballero C.J. (2023). *Bacillus thuringiensis* Cyt proteins as enablers of activity of Cry and Tpp toxins against *Aedes albopictus*. Toxins.

[3] Soares Figueiredo C., Nunes Lemes A.R., Sebastião I., Desidério J.A. (2019). Synergism of the *Bacillus thuringiensis* Cry1, Cry2, and Vip3 Proteins in *Spodoptera frugiperda* Control. Appl. Biochem. Biotechnol..

[4] Zhang Z., Yang X., Wang W., Wu K. (2024). Insecticidal effects of transgenic maize Bt-Cry1Ab, Bt-Vip3Aa, and Bt-Cry1Ab+Vip3Aa against the oriental armyworm, *Mythimna separata* [Walker] in Southwest China. Toxins.

[5] Chen S., Wang W., Kang G., Yang X., Wu K. (2024). Toxic effects of Bt-(Cry1Ab+Vip3Aa) maize on storage pest *Paralipsa gularis* (Zeller). Toxins.

[6] de Oliveira J.A., Negri B.F., Hernández-Martínez P., Basso M.F., Escriche B. (2023). Mpp23Aa/Xpp37Aa insecticidal proteins from *Bacillus thuringiensis* (Bacillales: Bacillaceae) are highly toxic to *Anthonomus grandis* (Coleoptera: Curculionidae) larvae. Toxins.

[7] Wei J., Lum A., Schepers E., Liu L., Weston R.T., McGinness B., Heckert M.J., Xie W., Kassa A., Bruck D. (2023). Novel insecticidal proteins from ferns resemble insecticidal proteins from *Bacillus thuringiensis*. Proc. Natl. Acad. Sci. USA.

[8] Jurat-Fuentes J.L., Heckel D.G., Ferré J. (2021). Mechanisms of Resistance to Insecticidal Proteins from *Bacillus thuringiensis*. Ann. Rev. Entomol..

[9] Wang Y., Zhao W., Han S., Wang L., Chang X., Liu K., Quan Y., Wang Z., He K. (2023). Seven years of monitoring susceptibility to Cry1Ab and Cry1F in asian corn borer. Toxins.

[10] Infante O., Gómez I., Pélaez-Aguilar A.E., Verduzco-Rosas L.A., García-Suárez R., García-Gómez B.I., Wang Z., Zhang J., Guerrero A., Bravo A. (2024). Insights into the structural changes that trigger receptor binding upon proteolytic activation of *Bacillus thuringiensis* Vip3Aa insecticidal protein. PLoS Pathog..

[11] Bravo A., Pacheco S., Gómez I., Soberón M., Jurat-Fuentes J.L. (2023). Chapter Two, Mode of action of *Bacillus thuringiensis* Cry pesticidal proteins. Advances in Insect Physiology.

[12] Fabrick J.A., Wu Y., Jurat-Fuentes J.L. (2023). Chapter Four, Mechanisms and molecular genetics of insect resistance to insecticidal proteins from *Bacillus thuringiensis*. Advances in Insect Physiology.

[13] Pacheco S., Chiñas M., Gómez J.U., Peláez-Aguilar A.E., do Nascimento N.A., Cantón P.E., Sánchez J., López-Molina S., Gómez I., Soberón M. (2025). ABC transporters knockout in *Aedes aegypti* induces upregulation of paralogous genes, avoiding resistance development to *Bacillus thuringiensis* Cry toxins. PLoS ONE.

[14] Kim S., Wang Y., Miyamoto K., Takasu Y., Wada S., Iizuka T., Sato R., Watanabe K. (2022). Cadherin BtR175 and ATP-binding cassette transporter protein ABCC2 or ABCC3 facilitate *Bacillus thuringiensis* Cry1Aa intoxication in *Bombyx mori*. J. Insect Biotechnol. Sericol..

[15] Wang X., Yue Y., Zhai Y., Wang F., Zhuang X., Wu S., Yang Y., Tabashnik B.E., Wu Y. (2025). Functional redundancy in the toxic pathway of Bt protein Cry1Ab, but not Cry1Fa, against the Asian corn borer. Proc. Natl. Acad. Sci. USA.

[16] Wang H., Bian H., Liu Z., Liu Y., Wang P., Liu K. (2025). Two pathways mediate toxicity of Cry1Ac in *Mythimna separata*: One is ABCC2-dependent and the other involves ABCC3-CAD interaction. Int. J. Biol. Macromol..

[17] Heckel D.G. (2025). Built-in redundant killing by Bt Cry1Ab toxin delays insect resistance. Proc. Natl. Acad. Sci. USA.

[18] López-Molina S., Guerrero A., Pacheco S., Wang Z., Zhang J., Sánchez J., Zavala G., Soberón M., Bravo A. (2025). Cry11Aa toxin of *Bacillus thuringiensis* interactions with intracellular organelles in insect gut implicating actin depolymerization, massive endocytosis, and vesicle secretion. Int. J. Biol. Macromol..

